# Crystallization dynamics and interface stability of strontium titanate thin films on silicon

**DOI:** 10.1107/S160057671500240X

**Published:** 2015-03-12

**Authors:** Florian Hanzig, Juliane Hanzig, Erik Mehner, Carsten Richter, Jozef Veselý, Hartmut Stöcker, Barbara Abendroth, Mykhaylo Motylenko, Volker Klemm, Dmitri Novikov, Dirk C. Meyer

**Affiliations:** a Institute of Experimental Physics, TU Bergakademie Freiberg, Leipziger Strasse 23, 09596 Freiberg, Germany; b Hamburger Synchrotronstrahlungslabor at Deutsches Elektronen-Synchrotron, Notke­strasse 85, 22607 Hamburg, Germany; c Institute of Materials Science, TU Bergakademie Freiberg, Gustav-Zeuner-Strasse 5, 09596 Freiberg, Germany; d Faculty of Mathematics and Physics, Charles University in Prague, Ke Karlovu 5, 121 16 Prague, Czech Republic

**Keywords:** strontium titanate, thin films, silicon substrates, crystallization dynamics, interface stability

## Abstract

Different physical vapor deposition methods have been used to fabricate strontium titanate thin films. Within the binary phase diagram of SrO and TiO_2_ the stoichiometry ranges from Ti rich to Sr rich, respectively. The crystallization of these amorphous SrTiO_3_ layers is investigated by *in situ* grazing-incidence X-ray diffraction using synchrotron radiation. The crystallization dynamics and evolution of the lattice constants as well as crystallite sizes of the SrTiO_3_ layers were determined for temperatures up to 1223 K under atmospheric conditions applying different heating rates. At approximately 473 K, crystallization of perovskite-type SrTiO_3_ is initiated for Sr-rich electron beam evaporated layers, whereas Sr-depleted sputter-deposited thin films crystallize at 739 K. During annealing, a significant diffusion of Si from the substrate into the SrTiO_3_ layers occurs in the case of Sr-rich composition. This leads to the formation of secondary silicate phases which are observed by X-ray diffraction, transmission electron microscopy and X-ray photoelectron spectroscopy.

## Abstract 2

Nonstoichiometric SrTiO_3_ thin films were fabricated by different thin-film deposition methods. The impact on the oxide/silcon interface stability as well as the crystallization onset temperature is investigated.

## 1. Introduction   

Metal–insulator–metal stacks are a promising concept for nonvolatile memories based on resistive switching (Sawa, 2008 ▶; Waser *et al.*, 2009 ▶). Perovskite-type transition metal oxides are favorable materials for the required thin insulating layers, because of their wide band gaps (Kahn & Leyende, 1964 ▶; Benthem *et al.*, 2001 ▶) and comparatively high dielectric constants (Samara, 1966 ▶), as well as mixed ionic and electronic conductivity (Baiatu *et al.*, 1990 ▶). Strontium titanate is studied for its potential in resistive random access memory applications (Sawa, 2008 ▶; Waser & Aono, 2007 ▶), since it exhibits a metal–insulator transition with a change in electrical resistance of over several orders of magnitude (Watanabe *et al.*, 2001 ▶; Beck *et al.*, 2000 ▶). Investigations of the resistive switching mechanisms and improvements of the device performance focus mostly on amorphous thin films (Kügeler *et al.*, 2011 ▶; Yan *et al.*, 2010 ▶; Jung *et al.*, 2010 ▶; Kang *et al.*, 2013 ▶; Liu *et al.*, 2013 ▶), because they offer lower leakage currents compared to polycrystalline layers which have grain boundaries as leakage paths and non-isotropic electrical properties (Wilk *et al.*, 2001 ▶). However, resistive switching behavior is also found in strontium titanate bulk crystals (Stöcker *et al.*, 2010 ▶; Wojtyniak *et al.*, 2013 ▶), as well as epitaxially grown and polycrystalline thin films (Szot *et al.*, 2007 ▶; Shibuya *et al.*, 2010 ▶; Menke *et al.*, 2009 ▶; Choi *et al.*, 2005 ▶; Sun *et al.*, 2011 ▶). Proposed mechanisms are governed by conductive filament formation resulting from redistribution of point defects. These are also known to influence the crystalline structure of the material (Hanzig *et al.*, 2013 ▶, 2015 ▶). Likewise, the valence state of titanium in strontium titanate switches reversibly under the influence of an external electric field (Leisegang *et al.*, 2009 ▶; Hanzig *et al.*, 2014 ▶). Therefore, the question arises, how do microstructure and crystallization influence the switching mechanism and stability in polycrystalline thin films? The starting point for such investigations is the equilibrium phase diagram of the SrO–TiO_2_ quasi-binary system (Levin *et al.*, 1964 ▶). At a composition of 50% SrO and 50% TiO_2_, SrTiO_3_ crystallizes in the cubic structure with space group 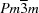. Stoichiometry deviations towards higher Sr content lead to the formation of the homologous series of Ruddlesden–Popper (RP) phases SrO(SrTiO3)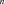 (Ruddlesden & Popper, 1957 ▶, 1958 ▶) with space group 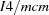. The RP phases are composed of perovskite unit cells that are shifted by [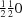] after *n* layers, thereby introducing an additional SrO plane. *Ab initio* calculations show that RP phases have electronic properties comparable to those of SrTiO_3_ (Zschornak *et al.*, 2010 ▶), but reveal tunable permittivity (Bhalla *et al.*, 2000 ▶) and band gap (Zschornak *et al.*, 2010 ▶). Ti excess in the phase diagram leads to the formation of TiO_2_ precipitates in an SrTiO_3_ matrix. The ternary phase diagram Sr–Ti–O (Tanaka *et al.*, 2003 ▶) predicts additional strontium titanate derived phases, *e.g.* Magneli phases (Andersson *et al.*, 1957 ▶), in the case of oxygen deficiency. For thin layers of a ternary oxide the microstructure and in turn the optical and electric properties do not depend solely on the given stoichiometry. Nucleation and growth of crystalline phases are also influenced by the substrate through lattice mismatch and roughness. Since SrTiO_3_ may be used as a high dielectric constant gate oxide in CMOS-based devices, the interfaces of these thin films in contact with silicon are still under extensive investigation. In particular, numerous experimental and theoretical studies on the stability of binary oxides (Hubbard & Schlom, 1996 ▶; Gutowski *et al.*, 2002 ▶), ternary oxides with perovskite structure (Goncharova *et al.*, 2006 ▶) and especially scandates on Si have been carried out (Sivasubramani *et al.*, 2006 ▶; Adelmann *et al.*, 2008 ▶; Copel *et al.*, 2010 ▶). At temperatures above 770 K, the thermal stability of the interface of the oxide thin film and silicon substrate decreases (El Kazzi *et al.*, 2007 ▶) and silicon interdiffusion interferes with the crystallization of the thin-film material system. The decomposition of ternary oxide thin films has been observed (Adelmann *et al.*, 2008 ▶), as well as cation diffusion into the substrate accompanied by silicate formation (Copel *et al.*, 2010 ▶). Even for epitaxial SrTiO_3_ on Si, extensive investigations regarding interface instability have been reported (Goncharova *et al.*, 2006 ▶; Delhaye *et al.*, 2006 ▶; Hu *et al.*, 2014 ▶).

Although stoichiometric SrTiO_3_ precursor material has been used in the present study, we show here that deviations from the ideal SrTiO_3_ stoichiometry are introduced already during the deposition process. Further, we report on the crystallization behavior of Sr-rich and Sr-deficient strontium titanate thin films during annealing under ambient atmosphere, including an *in situ* analysis of the growth kinetics of the cubic SrTiO_3_ phase in these films. Finally, special emphasis is put on the thermal stability of the interface between the transition metal oxide and silicon.

## 2. Materials and methods   

Strontium titanate thin films were prepared *via* electron beam evaporation (EBE) with an Edwards Auto 500 utilizing coarse-grained and ball-milled strontium titanate obtained from CrysTec GmbH, Berlin. Further fabrication of SrTiO_3_ thin films was done by radio frequency magnetron sputtering (RF SP) on a Bestec UHV magnetron sputtering system with argon plasma using a strontium titanate target (purity 99.95%) from Testbourne Ltd, England. All substrates were (001)-oriented monocrystalline Si wafers with a native oxide surface layer of approximately 2 nm thickness. For reasons of comparability no substrate heating was applied. Process parameters are summarized in Table 1 ▶.

The elementary composition of all thin-film samples was obtained from wavelength dispersive X-ray fluorescence (XRF) using a Bruker S8 Tiger spectrometer employing a rhodium source and LiF200, XS-55 and PET monochromator crystals, covering a spectral energy range from 60 keV down to 0.491 keV. A full fundamental parameter approach was adopted for all calculations, as implemented in the software *ML Quant* (Bruker AXS, Karlsruhe, Germany.). The Ti/Sr ratio of the material residues in the crucible after evaporation was checked by energy dispersive XRF (EDX) using a JEOL JSM-6400 scanning electron microscope equipped with a Noran EDX detector. Layer thicknesses were determined from spectroscopic ellipsometry (SE) with a Sopra GES 5E and X-ray reflectivity (XRR) using a Seifert HZG4. Layer density was evaluated from XRR with the *pyxrr* (Richter, 2014 ▶) analysis software. Depth-resolved stoichiometry analysis was performed with X-ray photoelectron spectroscopy (XPS) on a Thermo Fisher Scientific Escalab 250Xi. High-resolution transmission electron microscopy (HRTEM) was carried out using a 200 kV analytical high-resolution transmission electron microscope (JEOL JEM 2200 FS) equipped with an in-column Ω filter to improve image quality by removing inelastic scattered electrons. In addition, TEM imaging was employed for depth calibration of the XPS compositional profile. *In situ* temperature-dependent grazing-incidence X-ray diffraction (GI-XRD) was measured at beamline E2 of the DORIS III storage ring (HASYLAB at DESY, Hamburg). Measurements were carried out using monochromatic light of 12 480 eV energy (approximately 1 Å wavelength). The diffraction patterns were recorded at a constant 2θ detector angle of 15° with a Dectris Mythen one-dimensional photodiode array and an exposure time of 150 s, while the sample surface was inclined by an angle ω of 2.5° with respect to the incident beam. Sample heating was facilitated by an Anton Paar DHS 1100 oven with a carbon dome under atmospheric conditions and controlled by a Eurotherm 2604 temperature controller. Interfering carbon dome reflections were cleared from the diffraction patterns by a copper aperture, thus limiting the temperature to a maximum of 1223 K. Afterwards, angle calibration of the diffraction data was performed using GI diffraction data from a Philips X’PERT thin-film system (PW3020 goniometer, 0.4° equatorial collimator and planar Ge monochromator) with copper radiation in a 2θ range of 10–120°. Depth-resolved X-ray diffraction data were collected at glancing angles ω of 0.125–8.45° to obtain the distribution of crystalline phases within the samples. The development of the SrTiO_3_ phase properties during temperature evolution was determined by Rietveld analysis utilizing Bruker’s *TOPAS 4.2* software with a combined 1/*x* and sixth-order polynomial background. To enable convergence of the fit routine, secondary phases were taken into account as peak phases.

## 3. Results   

### 3.1. As-deposited strontium titanate thin films   

The layer stoichiometries, thicknesses and densities of the RF SP and EBE samples as determined from XRF, SE and XRR are summarized in Table 2 ▶. The RF magnetron sputtered sample shows strontium deficiency with an Sr/Ti ratio of 0.8. All thin films prepared by electron beam evaporation reveal a distinct excess of strontium with an Sr/Ti ratio of 1.9. Thus, the residue of the material in the evaporation crucible was analyzed by EDX. The results show that the residue is inhomogeneously depleted of Sr, with the Ti/Sr ratio approximately matching the Sr/Ti ratio of the EBE films. Both kinds of samples reveal similar densities of 3.70–3.85 g cm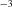, which is much lower than for single-crystal strontium titanate with a density of 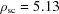
 g cm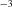 (Abramov *et al.*, 1995 ▶). As characterized by XRD, all as-prepared layers are amorphous.

### 3.2. *In situ* crystallization   

**Figure 1:**
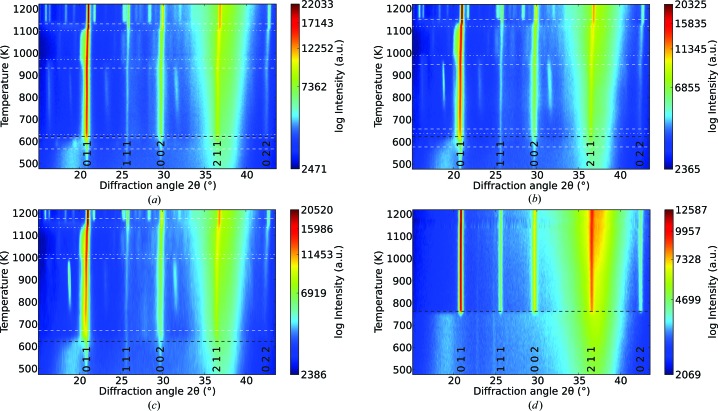
Crystallization of SrTiO_3_ thin films monitored by GI-XRD through a temperature ramp of (*a*) 2 K min^−1^, (*b*) 4 K min^−1^ and (*c*) 8 K min^−1^ for Sr-rich (EBE) and (*d*) 4 K min^−1^ for Sr-deficient (RF SP) layers. The diffraction angle is shown for 12 480 eV photon energy. The appearance and disappearance of additional reflections are highlighted by white dashed and dash–dot lines, respectively. The black dashed line indicates the vanishing amorphous halo. Reflections of SrTiO_3_ are labeled.

**Figure 2:**
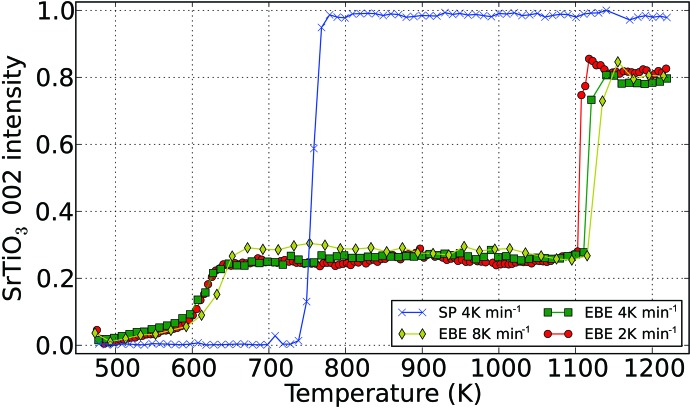
The evolution of the 002 reflection intensity with increasing temperature as extracted from the Bruker *TOPAS* SrTiO_3_ *hkl* output files. Intensities are scaled with respect to layer thicknesses and the global count maximum.

**Figure 3:**
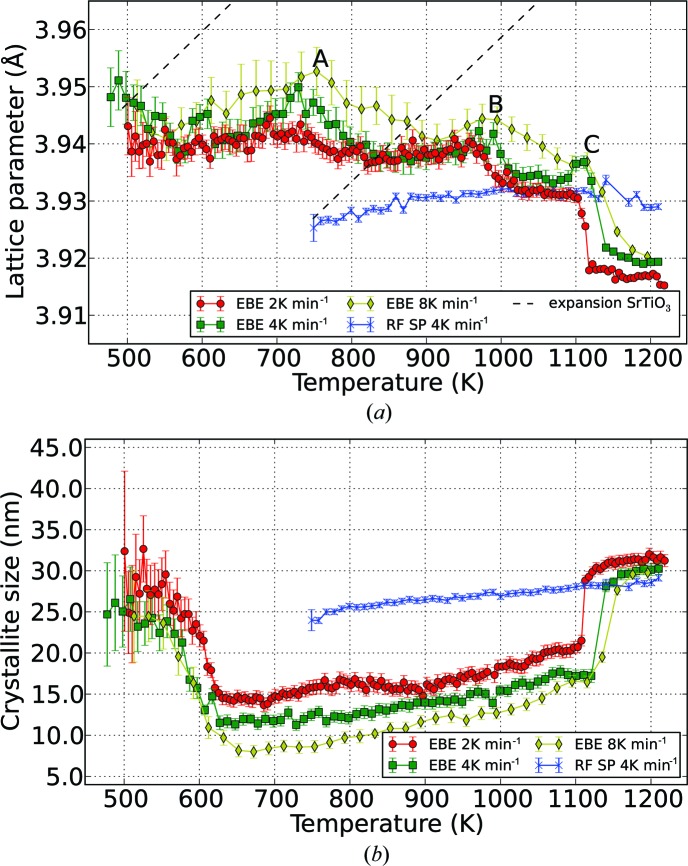
Temperature dependence of (*a*) lattice parameter and (*b*) crystallite size of SrTiO_3_ extracted from *in situ* GI-XRD with different temperature ramps of 2, 4 and 8 K min^−1^ for Sr-rich (EBE) and 4 K min^−1^ for Sr-depleted layers (RF SP), determined by Rietveld refinement using *TOPAS*. Dashed lines highlight the relative thermal lattice expansion calculated from the SrTiO_3_ thermal expansion coefficient (de Ligny & Richet, 1996 ▶). Prominent points in the development of the EBE samples’ crystallization are highlighted by the letters A, B and C.

The EBE samples were heated at rates of 2, 4 and 8 K min^−1^, whereas only the rate of 4 K min^−1^ was used to crystallize the RF SP sample. Fig. 1 ▶ shows the *in situ* GI-XRD data of EBE- and RF-prepared samples, subjected to different temperature ramps. Reflections attributed to the cubic SrTiO_3_ phase are indicated in these graphs. The broad reflection present at a 2θ angle of 36.8° in all patterns is due to the silicon substrate [*Umweganregung* of the Si 311 reflection (Többens *et al.*, 2001 ▶)]. In the EBE samples, superimposed on the amorphous background, weak reflections of the cubic SrTiO_3_ phase (Abramov *et al.*, 1995 ▶) appear shortly after starting the X-ray diffraction measurement at 473 K. Despite the different heating rates, all EBE samples show comparable crystallization behavior. Starting with the vanishing of the amorphous background (see black dashed lines in Fig. 1 ▶), the intensities of the reflections of the cubic SrTiO_3_ phase first increase rapidly. This stage is followed by a plateau of constant intensity that is accompanied by the consecutive emergence and disappearance of additional phases (see white dashed and dash–dot lines in Fig. 1 ▶). Fig. 2 ▶ summarizes the intensity evolution of the SrTiO_3_ 002 reflection for all annealing experiments. At elevated temperatures, a narrow temperature window arises, displaying strong reflections exclusively from the cubic strontium titanate phase. Above the latter temperature range, the diffraction patterns show similar reflections attributed to secondary phases. The deviations in onset and dissolution temperature of the additional phases correlate with the particular magnitude of the heating rate (see Table 3 ▶). This formation and dissolution of secondary phases also influences the lattice parameter and crystallite size of cubic SrTiO_3_ (see Figs. 3 ▶ *a* and 3 ▶ *b*). Analysis of the respective SrTiO_3_ reflection intensities shows no deviations from the theoretical intensity distribution, thus indicating randomly oriented crystallites. In the case of the RF SP sample, cubic SrTiO_3_ initially crystallizes at 739 K. Here the reflections from SrTiO_3_ reach their maximum in intensity at a temperature of 763 K (see Fig. 2 ▶), with the vanishing of the amorphous background. In the entire temperature range, the diffraction pattern of the RF SP sample contains two very weak signals at 2θ values of 22.9 and 24.9°, which are assignable neither to SrTiO_3_ nor to the experimental setup of the beamline, because they are present in *ex situ* X-ray diffraction patterns too. The logarithmic scaling emphasizes the signals which are almost indistinguishable from the background in individual diffraction patterns. In order to increase the signal-to-noise ratio for phase matching and indication, diffraction patterns were summed through the whole temperature range. Unfortunately, phase matching was unsuccessful. However, a few expected phases like rutile and anatase were excluded with certainty.

Details of the temperature dependence of the lattice parameter *a* and crystallite size were obtained by a basic Rietveld analysis with Bruker *TOPAS*, starting at the crystallization temperature for the respective layers. The thin film prepared by RF magnetron sputtering reveals an initial lattice constant of 3.925 Å at 739 K, whereas for the EBE samples it is 3.945 Å. In the measured temperature range, the RF SP sample lattice parameter stays nearly constant. In contrast, those of the EBE samples undergo strong alteration at temperatures up to 1173 K, while additional phases form and disappear. All samples show lattice parameters larger than those reported for pure SrTiO_3_, with *a* = 3.905 Å (Abramov *et al.*, 1995 ▶), which can be attributed to deviations of the Sr/Ti ratio from 1.0 (Brooks *et al.*, 2009 ▶). The low density found for all samples is mainly due to microporosity. Focusing on the temperature intervals where only SrTiO_3_ reflections are detected in the EBE diffraction patterns (compare pure SrTiO_3_ in Table 3 ▶) a distinct drop of the lattice parameter is observed (see Fig. 3 ▶ *a*), while at the same time the crystallite size increases (see Fig. 3 ▶ *b*). At the crystallization temperature the RF SP sample crystallite size is about 24 nm and grows steadily with increasing temperature up to 29 nm (see Fig. 3 ▶ *b*). Among the EBE thin films the crystallite size evolves similarly. Their initial and final crystallite sizes vary from 29 to 25 nm and 32 to 30 nm, respectively. Fig. 3 ▶(*b*) indicates a drop of the size of the SrTiO_3_ crystallites with the appearance of additional phases. The inverse behavior can be seen at temperatures where additional phases disappear. The final crystallite sizes are of comparable magnitude, independent of the fabrication method. For all EBE samples the irreversible formation of at least one secondary phase is observed above 1132.7, 1150.2 and 1175.2 K, respectively.

### 3.3. Secondary phase investigation   

**Figure 4:**
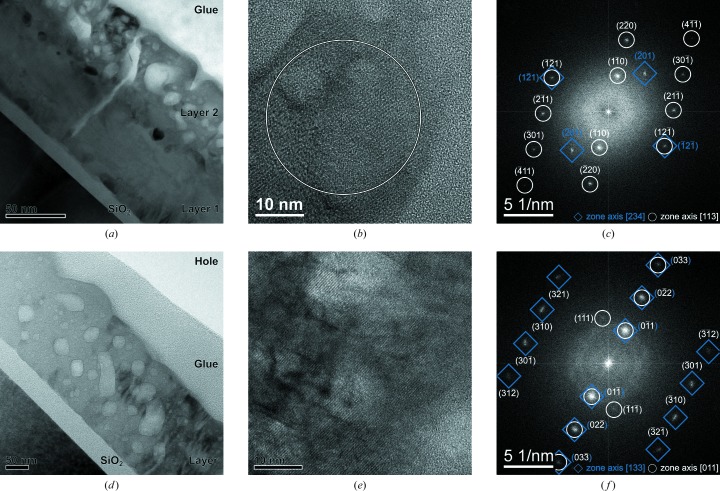
(*a*) TEM cross section of the EBE sample at 40k magnification, showing a segregation into two different layers. (*b*) HRTEM image (400k magnification) of the top layer with a circle highlighting the crystalline size determined from the X-ray diffraction method. (*c*) Respective fast Fourier transform showing reflections, with squares indicating the [234] and circles displaying the [113] orientation of the cubic SrTiO_3_ phase. (*d*) TEM cross section of the RF SP sample with 100k magnification, displaying one layer. (*e*) HRTEM image at 400k magnification, highlighting the crystalline areas. (*f*) Fast Fourier transform showing cubic SrTiO_3_ in the [133] and [011] orientations, marked with squares and circles, respectively.

**Figure 5:**
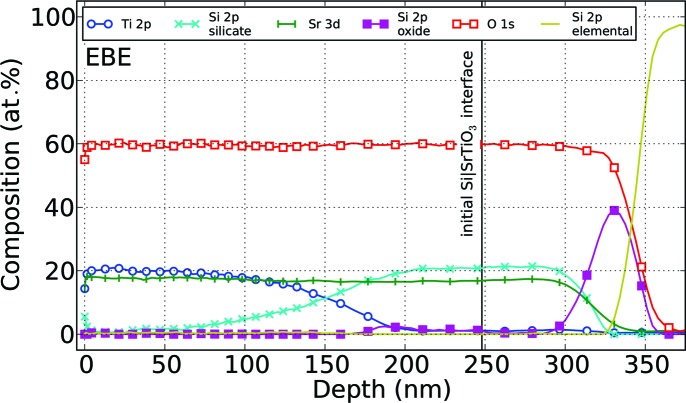
Depth-resolved XPS measurement of an Sr-rich SrTiO_3_ (EBE) thin film crystallized using a heating rate of 2 K min^−1^ up to 1223 K. The depth axis is calibrated according to Fig. 4 ▶(*a*).

**Figure 6:**
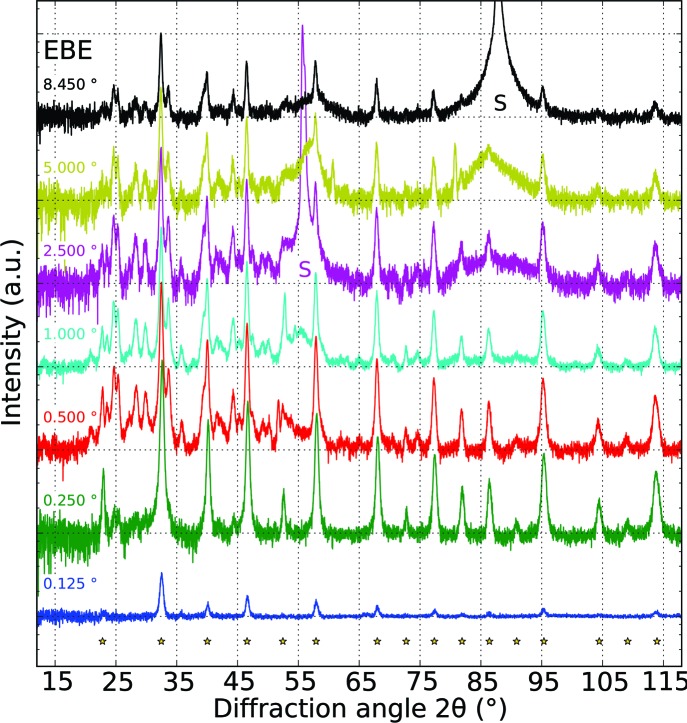
Depth-resolved gracing-incidence X-ray diffraction of an Sr-rich SrTiO_3_ (EBE) thin film (Cu radiation). The related glancing angles are indicated on top of each diffraction pattern. Reflections from cubic SrTiO_3_ (Abramov *et al.*, 1995 ▶) are labeled with stars and those from the substrate (Többens *et al.*, 2001 ▶) by the letter S.

The formation of additional phases during crystallization motivates a closer examination of the thin films with HRTEM. Fig. 4 ▶ displays the cross-section images of an electron beam evaporated thin film (*a*) in comparison to an RF-sputtered sample (*d*) after annealing up to 1223 K. Whereas the RF SP sample exhibits one well defined layer, the EBE sample is inhomogeneous, with at least two distinct layers on top of the substrate, which was oxidized in the formation process. To obtain a compositional profile of an EBE sample, depth-resolved photoelectron spectroscopy was employed, averaging over a sample area of 1.13 mm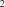 (see Fig. 5 ▶). As with HRTEM, a decomposition into two layers of 165 and 140 nm thickness was detected, exhibiting Sr:Ti:O and Si:Sr:O ratios of 1:1:3. Within the topmost layer, HRTEM micrographs at a magnification of 400k show crystalline areas in both EBE and RF SP samples (Figs. 4 ▶ *b* and 4 ▶ *e*). Reflections in the respective Fourier transformation are assigned to the cubic room-temperature phase of strontium titanate (Fig. 4 ▶ *c* and 4 ▶ *f*). Besides the stoichiometry, in the additional layer the Si 2*p* electron binding energies were determined to be 102 eV, indicating the formation of silicates (Shutthanandan *et al.*, 2002 ▶). Underneath, a silicon oxide film has developed during annealing, with Si 2*p* binding energies of 99.5 and 104.0 eV, typical for SiO_2_ bonding (Shutthanandan *et al.*, 2002 ▶). In combination, these measurements give evidence of silicon diffusion into the Sr-rich oxide layer, leading to an increase of the film thickness up to 305 nm with homogeneous content of oxygen and strontium across the uppermost layers. To complement the extremely local TEM images with volume average information and to identify the depth distribution of secondary phases, angle-resolved GI-XRD was conducted (see Fig. 6 ▶) at glancing angles ranging from 0.125 to 8.450°, which correspond to a penetration depth of 2.95 (2.93) nm to 3.61 (5.41) µm in SrTiO_3_ (SrSiO_3_) when using copper radiation and the densities listed in Table 2 ▶. The ω value 8.45° corresponds to an attenuation length in the material of the Cu radiation equivalent to synchrotron radiation with the glancing angle of 2.5° used at beamline E2. For ω = 0.125° only the very near surface region is probed. Here exclusively reflections of the pure SrTiO_3_ are detected. The fact that additional phases appear in deeper regions coincides with results drawn from XPS measurements. Owing to *Umweganregung*, the 311 and 422 reflections from the Si substrate (Többens *et al.*, 2001 ▶) are visible at 2θ values of approximately 56 and 88°.

## 4. Discussion   

In spite of the use of stoichiometric precursor materials, different physical vapor deposition techniques nevertheless result in thin films with deviating stoichiometry within the binary system SrO and TiO_2_. Their composition was determined to be either Sr-deficient Sr_4_Ti_5_O_18_ or Sr- and O-rich Sr_2_TiO_7_ (see Table 2 ▶), which clearly indicates the different impact of the deposition method on film stoichiometry. Referring to the binary phase diagram of SrO and TiO_2_ (Levin *et al.*, 1964 ▶), samples should crystallize in the form of cubic SrTiO_3_ with segregation of TiO_2_ or Ruddlesden–Popper phases (Ruddlesden & Popper, 1957 ▶, 1958 ▶), respectively. In the case of Sr-rich layers from electron beam evaporation the deviation is attributed to preferential ablation of SrO due to its lower evaporation enthalpy from the SrTiO_3_ melt compared to TiO_2_ (Dam *et al.*, 1996 ▶). In contrast, the presence of Sr-depleted layers resulting from magnetron sputtering is attributed to preferential sputtering of the stoichiometric SrTiO_3_ target. Because of the comparable atomic masses of Ar and Ti as compared to Sr, the latter is sputtered less effectively.

**Figure 7:**
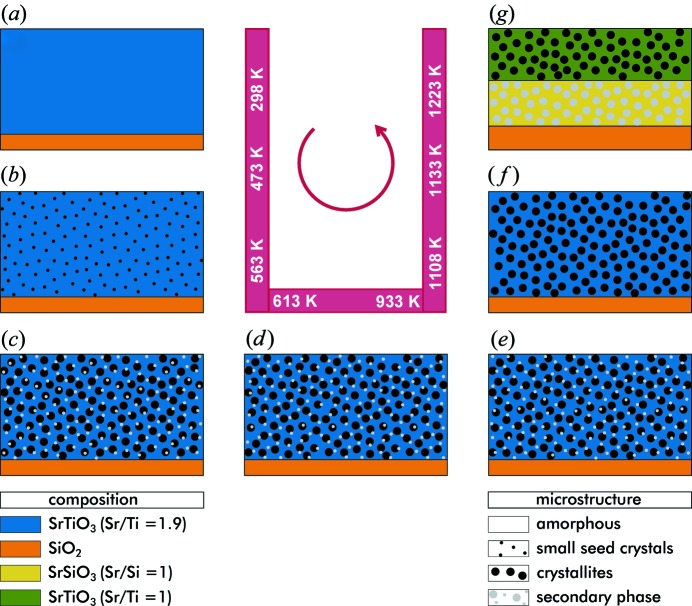
Sketch of the supposed evolution of the crystallization for Sr-rich samples (EBE): (*a*) as-deposited amorphous layer with an Sr/Ti ratio of 1.9, (*b*) growth of SrTiO_3_ seed crystals, (*c*) an enlarged number of SrTiO_3_ crystallites accompanied by the first secondary phase, (*d*) and (*e*) appearance of alternative secondary phases, whereas the former phase vanishes, (*f*) presence of crystallites from cubic SrTiO_3_ only, and (*g*) separation into two distinctive layers comprising SrTiO_3_ and SrSiO_3_, respectively. The amorphous SiO_2_ layer thickness increases. All temperatures are exemplarily extracted from the sample with 2 K min^−1^ heating rate.

For clarity, Fig. 7 ▶ depicts the crystallization behavior of Sr-rich samples during annealing up to 1223 K. The background intensity in the XRD data is related to the presence of an amorphous phase (Fig. 7 ▶ *a*). Its dissolution during annealing coincides with increasing structural ordering in the layers. Hence the variation in the temperature at which this background intensity vanishes is attributed to different degrees of structural and compositional ordering in the as-deposited layers. Consistently, an even higher degree of chemical order in the as-deposited layer can be achieved by using atomic layer deposition, which further increases the crystallization temperature (Rentrop *et al.*, 2015 ▶). Because of the combination of low particle energy and inhomogeneous evaporation, only a randomly distributed fraction of the EBE layers’ volume exhibits stoichiometry close to SrTiO_3_. Here the crystallization of the cubic SrTiO_3_ phase starts almost at the beginning of the temperature treatment, leaving a strontium-oxide-rich matrix in the surrounding region (see Fig. 7 ▶ *b*). Owing to the small peak width, Rietveld refinement results in relatively large crystallites, and the weak peak intensities suggest a low diffracting volume of these seed crystallites, leading to a large error in the determination of the particle size. The fact that the initial lattice parameter is larger than the reported value of the cubic SrTiO_3_ phase (Abramov *et al.*, 1995 ▶) suggests a nonstoichiometry either to the Sr- or to the Ti-rich side (Brooks *et al.*, 2009 ▶). Further increase of the annealing temperature to approximately 573 K allows successive crystallization of several phases from the initially amorphous matrix (see Fig. 1 ▶ *a*–1 ▶ *c*), causing a drop in crystallite size (Fig. 1 ▶ *b*). The tabulated (de Ligny & Richet, 1996 ▶) and observed coefficient of thermal expansion do not match (see Fig. 3 ▶ *a*). In detail, the rapid changes in lattice parameter marked A, B, and C in Fig. 3 ▶(*a*) correspond to the formation of secondary phases (see Fig. 7 ▶ *c*–7 ▶ *e*). Since the stoichiometry of the final SrTiO_3_ is nearly ideal we assume that the lattice expansion is governed by the relaxation of nonstoichiometry (Brooks *et al.*, 2009 ▶). In contrast, the cation nonstoichiometry in the SrTiO_3_ crystallites has to remain constant if the lattice parameter rises as the temperature approaches the prominent points A, B and C in Fig. 3 ▶(*a*). In terms of thermodynamics the final state is probably still not fully equilibrated because of the unusually low lattice constant for this temperature.

At elevated temperatures two processes have to be discussed. First, the oxygen and strontium mobility is large enough to precipitate Sr and O at the interface to the substrate (see Fig. 4 ▶ *a*), leaving an approximately 100 nm-thick layer composed of phase-pure SrTiO_3_ on top (see Fig. 5 ▶). In between, an intermixing zone with gradients of titanium and silicon is thereby created. Second, SrTiO_3_ as well as SrO on Si are both reported to be thermodynamically unstable at higher temperatures (Hubbard & Schlom, 1996 ▶; Reiner *et al.*, 2010 ▶; El Kazzi *et al.*, 2007 ▶). Therefore, a reaction of excess Sr with SiO_2_ to form SrO by degradation of the SiO_2_ layer on top of the substrate is possible, and even in lower-temperature regions the presence of SrTiO_3_, SrO and Si can lead to formation of SrSiO_3_ (Hubbard & Schlom, 1996 ▶). In the case of strontium oxide, it is reported that the conversion of SrO at the interface with Si at higher temperatures first induces Sr_2_SiO_4_ formation, prior to the latter’s conversion to a phase close to SrSiO_3_ as the temperature is further increased (El Kazzi *et al.*, 2007 ▶). Furthermore, the surface of the Si substrates is oxidized by oxygen originating from the thin film, increasing the SiO_2_ layer thickness to approximately 25 nm as determined by XPS and TEM, starting at an initially natural silicon oxide layer. Possibly, the oxygen stems from the surrounding atmosphere as well. The observed growth of SiO_2_ is consistent with the prediction of the basic oxidation model proposed by Deal & Grove (1965 ▶). Subsequently, a diffusion of silicon from the substrate establishes the newly formed silicate layer (Fig. 7 ▶ *g*). Here, the XPS compositional profile between 200 and 300 nm from the layer surface indicates Si reaching a homogeneous content of 20%, framed by a decreasing percentage towards SrTiO_3_ as well as SiO_2_ (see Fig. 5 ▶). However, we were unable to match impurity phases to entries of the ternary system Sr:Si:O in the common powder pattern databases. As the Sr-deficient thin film shows no additional crystalline phases in the diffraction pattern up to 1223 K without a visible decomposition in the TEM cross section, it is concluded that the additional titanium stabilizes SrTiO_3_ on top of the silicon substrate.

## 5. Conclusion   

We have reported the preparation of initially amorphous SrTiO_3_ thin films by electron beam evaporation and RF magnetron sputtering. *In situ* X-ray diffraction of Sr-rich and Sr-depleted layers during annealing in air up to 1223 K was performed using synchrotron light. Clear differences in the crystallization onset of cubic SrTiO_3_ have been observed. During heat treatment, electron beam evaporated samples exhibit a number of unknown secondary phases, and transmission electron microscopy confirms the formation of an additional layer between the SrTiO_3_ film and the Si substrate. By means of X-ray photoelectron spectroscopy the composition was evaluated to be SrSiO_3_. For RF-sputtered samples the interface to the substrate is chemically stable and solely the cubic SrTiO_3_ phase is formed during annealing. The reactivity at the interface between substrate and thin film as well as formation of additional phases resulting from silicon interdiffusion is triggered by stoichiometry deviations and thus by the preparation method.

## Figures and Tables

**Table 1:** Process parameters

Parameter	EBE	RF SP
Base pressure (Pa)	5.0 10^3^	10^5^
Process gas pressure (Pa)		0.76
Beam current (mA)	50	
Process power (W)	239	150
Coating time (s)	600	7600
Maximum temperature (K)	340	302
Deposition rate (nm s^1^)	0.242	0.017

**Table 2:** Properties of amorphous as-deposited thin films obtained from XRF, SE and XRR

Sample	Ti (at.%)	Sr (at.%)	O (at.%)	Sr/Ti	Thickness (nm)	Density (gcm^3^)
EBE						
2Kmin^1^	10.7	20.1	69.1	1.9	248	3.75
4Kmin^1^	10.8	20.7	68.5	1.9	248	3.70
8Kmin^1^	10.9	20.4	68.7	1.9	248	3.73
Precursor	20.2	19.6	60.2	1.0		5.12

RF SP						
4Kmin^1^	18.9	15.3	65.8	0.8	97	3.83
Precursor	99.95% SrTiO_3_ †					

† From datasheet provided by manufacturer.

**Table 3:** Summary of secondary phase formation in particular temperature regimes during *in situ* XRD

Sample	EBE			RF SP
Parameter	2Kmin^1^	4Kmin^1^	8Kmin^1^	4Kmin^1^
Crystallization (K)	480.7	477.7	473.2	738.7
Amorphous until (K)	623.2	623.2	623.2	763.2
				
Secondary phases				
1st (K)	565.7630.7	577.2637.2		
2nd (K)	615.7972.2	658.2989.7	672.21114.7	
3rd (K)	932.21102.7	949.21120.7	994.71135.2	
Pure SrTiO_3_	1107.71127.2	1130.21140.2	1155.2	
Silicates (K)	>1132.7	>1150.2	>1175.2	
